# hsa_circ_0071106 can be used as a diagnostic marker for type 2 diabetes

**DOI:** 10.7150/ijms.52575

**Published:** 2021-04-03

**Authors:** Zheng Yingying, Yu Yongji, Cheng Qiuting, Liao Rifang, Zeng Zhuanping

**Affiliations:** 1 School of Public Health, Guangdong Pharmaceutical University, Guangzhou 510310, China.; 2 Department of Pharmacy, Sun Yat-Sen Memorial Hospital, Sun Yat-sen University, Guangzhou 510120, China.; 3 The Second People's Hospital of Huadu District, Guangzhou 510320, China.

**Keywords:** diagnostic marker, type 2 diabetes, circRNA microarray

## Abstract

We explored hsa_circ_0071106 as a diagnostic marker for type 2 diabetes (T2DM) in south China, and predicted the functional mechanism of the target circRNA. A total of 107 T2DM patients and 107 healthy reference persons were included as the research objects. In the first stage, the circRNA microarray was used to detect the peripheral blood of 4 T2DM and 4 control groups to screen the differential expression profile of circRNA. In the second stage, four circRNAs were screened from the differential expression profiles of circRNA, and real-time polymerase chain reaction (Q-PCR) technology was used to verify the blood samples of 103 T2DM and 103 controls. Finally, Gene Ontology (GO) and Kyoto Encyclopedia of Genes and Genomes (KEGG) pathway analysis in bioinformatics were used to predict the functional mechanism of target circRNA. Lastly, we found that hsa_circ_0071106 increase the risk of T2DM (OR=2.819 (95% CI: 1.415~5.616)). The area under the ROCcurve hsa_circ_0071106 was 0.690, the sensitivity was 62.1%, and the specificity was 69.9%. The function prediction results showed that hsa_circ_0071106 was involved in biological processes such as protein binding, gene transcription, and may be involved in the pathway of hsa-miR-29a-5p regulating diabetes, hsa_circ_0071106 may be used as a diagnostic marker for T2DM.

## Introduction

According to the International Diabetes Federation (IDF) Diabetes Atlas (9^th^ Edition, 2019), nearly 463 million diabetic patients exist worldwide, 1 in 2 adults with diabetes are undiagnosed 1. By 2040, the number of patients with diabetes may increase to 642 million 2,3. In the advanced stages of T2DM, patients often experience various complications, and it will also bring a very heavy financial burden 4. Therefore, early diagnosis and intervention are urgently needed. There are currently three methods for diagnosing diabetes. The oral glucose tolerance test (OGTT) is the gold standard for clinical detection of T2DM 5, but the process is time-consuming and complicated, fasting blood glucose (FPG) detection method is convenient, but its error rate is high 6, the detection of glycated hemoglobin (HbA1c) has not been standardized in China. Therefore, current diagnosis measures for T2DM show various insufficiencies, the above methods are considered for diagnosis and treatment when the suspect is suspected of having T2DM. When the patient is confirmed as T2DM, The critical period of abnormal glucose tolerance that can reverse diabetes has been missed 7. So it is necessary to explore early biomarkers with high specificity, high sensitivity and fast test speed for T2DM, which is conducive to the early prevention and treatment of T2DM.

With the maturation of molecular biology technologies such as high-throughput sequencing and the implementation of the Human Genome Project. In recent years, circRNA has been found to be a diagnostic marker for various diseases such as the cardiovascular system 8-9, nervous system 10-11 and tumors 12-13. CircRNA has the characteristics of structural stability 14, tissue specificity 15, evolutionary conservatism 16, and it is difficult for exonuclease to degrade it 17, and has sponge-like adsorption to inhibit the activity of microRNA 14, regulate gene transcription 18, bind RNA binding protein, participate in translation protein and other functions 19, these characteristics and functions have contributed to circRNA as a new biomarker and the target of disease treatment for the diagnosis of noninfectious chronic diseases such as diabetes. Therefore, in this study, the circRNA microarray analysis is used to screen out the significantly different circRNA expression profiles in T2DM, then the Q-PCR technology is used to verify reliability in the expanded population, and finally the target circRNA is analyzed through bioinformatics to predict the target genes and possible signal pathways related to T2DM, and provide a basis for the prevention and control measures of T2DM.

## Research design and methods

### Research object

The research object of this study comes from the healthy screening population over 18 years of age in Huadu District, Guangzhou, from January to October 2019. According to the 1:1 individual matching case control study design, the matching conditions are the same gender and community and age less than 5 years old, a total of 107 diabetic patients and 107 healthy controls were included. In the first stage, 4 pairs of samples were selected for circRNA microarray test to screen for differential expression profiles. In the second stage, 103 pairs of samples were taken for Q-PCR verification. And the Ethics Committee of Guangdong Pharmaceutical University approved the study.

### Inclusion criteria

#### Diabetes group

According to the “China T2DM Prevention and Treatment Guidelines (2017 Version)” 20 standard grouping: fasting blood glucose value ≥7.0 mmol/L or 2 h postprandial blood glucose value ≥11.1 mmol/L or glycated hemoglobin HbA1c ≥6.5% or a previous history of diabetes or have typical symptoms of diabetes such as multiple drinks, polyphagia, polyuria and unexplained weight loss, with random blood glucose values ≥ 11.1 mmol/L.

#### Control group

Healthy people with fasting blood glucose value <7.0 mmol/L and random blood glucose value <11.1 mmol/L and 2 hours postprandial blood glucose value <11.1 mmol/L in the same sex and same area and 5 years old as diabetic patients were selected as the control group.

### Exclusion criteria

Diabetes group and control group excluded patients with other types of diabetes such as type 1 diabetes, gestational diabetes, and patients with cerebral infarction, myocardial infarction, malignant tumors, and chronic inflammation.

### Whole blood sample collection and total RNA extraction

The subjects collected 3mL of whole blood using a vacuum blood collection tube containing EDTA anticoagulant in the morning on an empty stomach. Immediately freeze the samples and transport them to the ultra-low temperature refrigerator through dry ice to store them for subsequent sample extraction. RNAiso Blood (Takara Bio Inc, Japan) extraction reagent was used to extract total RNA from 300 ul of whole blood, Nano Drop-2000 was used to determine the concentration and purity of total RNA, and the integrity of total RNA was using modified agarose gel electrophoresis for detection.

### CircRNA microarray analysis

During the experiment, Arraystar Human circRNA Arrays v2 provided by Arraystar was used to extract the peripheral blood of 4 diabetes and 4 control groups for microarray analysis. The circRNA microarray used ribonucleic acid R (Rnase R) to decompose the total RNA into linear RNA and circular RNA, removed the linear RNA in the sample, enriched the circular RNA in the sample. Then the enriched circRNAs were amplified according to the random primer method and transcribed into cRNA with a fluorescent substance label, and then sequentially hybridized to the circRNA microarray according to the principle of nucleic acid hybridization. After the hybridization, the position of the reaction point and the fluorescence intensity are scanned using a chip scanner, and then imported into the relevant analysis software for data analysis. Differentially expressed circRNAs between two samples were identified through Fold Change, |FC|≥1.5 and *t* test, *P*<0.05 filtering.

### Q-PCR validates differentially expressed circRNAs

Four circRNAs were selected from circRNA differential expression profiles for population Q-PCR experiments. Both the Prime Script™ RT reagent Kit with gDNA Eraser and Q-PCR kits were equipped with Takara Bio Inc, Japan. Reverse transcription reaction system: 5×gDNA Eraser Buffer 2.0ul, gDNA Eraser 1.0 ul, add appropriate amount of total RNA and make up to 10ul with enzyme-free water, place at 42 °C and heat for 2 minutes to remove the DNA from the genome, then add to the above liquid PrimeScript RT Enzyme Mix I 1.0 ul, RT Primer Mix 1.0 ul, 5× PrimeScript Buffer 2 (for Real Time) 4.0 ul, RNase Free dH_2_O 4.0 ul, shake gently and immediately put at 37 °C for 15 minutes. The extension reaction was carried out under the conditions of reverse transcription inactivation in an environment of 85 °C and 5 seconds, and finally it was cooled under the heat preservation condition of 4. Q-PCR reaction system: TB Green Premix Ex Taq (Tli RNaseH Plius) (2X) 10 ul, PCR Forward Primer (10M) 0.8 ul, PCR Reverse Primer (10 M) 0.8 ul, ROX Reference Dye (20X) 0.4 ul, enzyme-free water 6 ul, cDNA 2 ul. Reaction conditions: 95 °C, 34 s cycle to activate enzyme activity once, 95 °C, 5 s, 60 °C, 34 s for 40 cycles of extension reaction, using housekeeping gene GAPDH as internal reference, and finally set the melting curve analysis. Table 1 is the primer sequence of target circRNA and GAPDH.

### Statistical analysis

The collected basic data and Q-PCR experimental data were integrated into the software SAS 9.4 for analysis. When the quantitative data presents the normal distribution of the data, the means ± standard deviation is used to represent, and the continuity data that does not present the normal distribution is represented by the median number and the quarter-value interval. The difference analysis of quantitative data between two groups was by paired *t* test or Wilcoxon signed-rank test, and the distribution difference of demographic characteristics and life behavior factors between diabetes group and control group was by paired χ^2^ test. The diagnostic value of circRNA in diabetes needs to be evaluated using the ROC curve analysis, calculating the area under the curve (AUC), sensitivity, specificity, maximum Youden index and the best cut-off point. The AUC<0.5 has no diagnostic value and AUC>0.5 has diagnostic value. To further analyze the relationship between target circRNA and diabetes, a multifactor conditional logistic regression model with correction for confounding factors was used for analysis.

## Results

### CircRNA microarray analysis results

**Figure 1:**
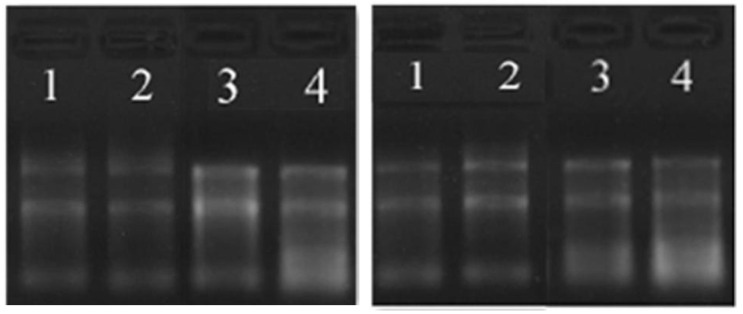
RNA agarose gel electrophoresis (the left picture is the diabetes group, the right picture is the control group).

The total RNA concentration in the peripheral blood of 4 pairs of diabetic group and the control group was 160~550 ng/ul, and the purity was between 1.8~2.3. The total RNA quality met the requirements of the circRNA microarray analysis. The modified agarose gel electrophoresis was used to detect the integrity of the total RNA. The 28s and 18s bands of the total RNA were clear, and the 5s band was vague, indicating that there was no obvious non-specific amplification band and primer dimer in the product band (Figure 1).

### Volcano and scatter plots

**Figure 2:**
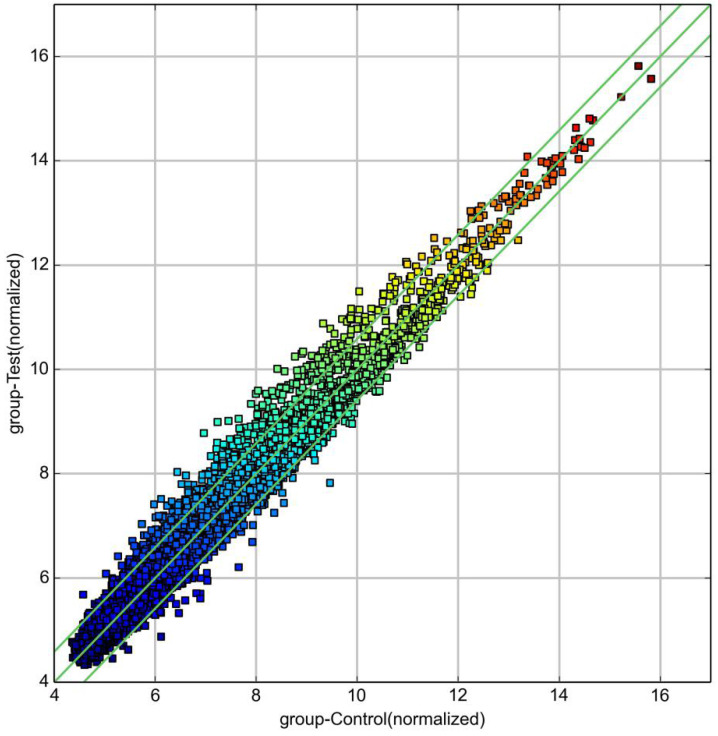
circRNA scatter plot (diabetes group and control group).

The log value of the log_2_ logarithmic conversion of the average expression of circRNA in the control group was plotted on the abscissa, and the log value of the log2 logarithmic conversion of the average expression of circRNA in the diabetes group was plotted on the ordinate (Figure 2). In the figure, the part above the line1 and below the line2 was |FC|≥1.5, P<0.05, the significant difference in expression of circRNA. Above line 1 was up-regulated circRNA, and below line 2 was down-regulated circRNA.

**Figure 3:**
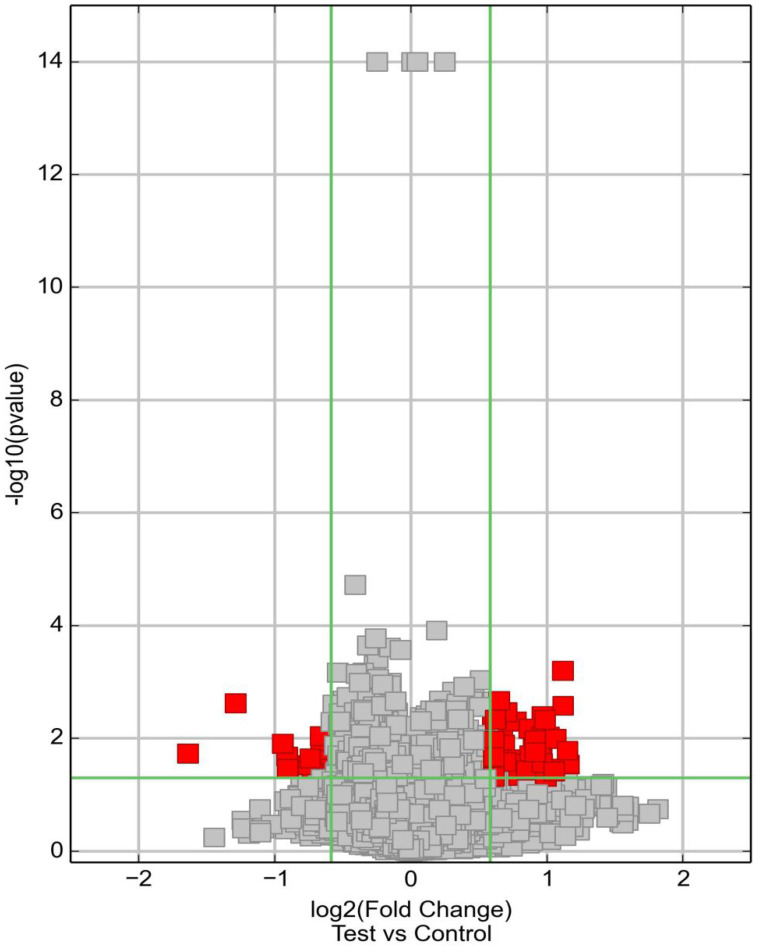
circRNA volcano graph (diabetes group and control group).

The volcano graph was plotted according to the FC and *P* value. The abscissa was log2 (FC) and the ordinate was -log10 (*P* value) to create a volcano graph (Figure 3). In the figure, the part above the horizontal green line and beyond the two vertical green lines indicated the distribution of circRNA with |FC| ≥1.5 and P<0.05. The red dots in the figure indicated the differential circRNA, some in the upper left corner indicated the down-regulated circRNA, and those in the upper right corner indicated the up-regulation circRNA.

### CircRNA differential expression profile

A total of 798 circRNAs were screened from the circRNA microarray results, of which 296 were up-regulated and 502 were down-regulated. However, according to the |FC| ≥1.5 and* P*<0.05, the results showed that a total of 86 circRNAs was differential expression, of which 70 expressions were up-regulated and 16 were down-regulated. The candidate biomarkers were selected from the 86 circRNAs utilizing stricter screening criteria: P<0.01, |FC| ≥2, exon and the original intensity is greater than 200. Four candidate circRNAs met these criteria: hsa_circ_0071106, hsa_circ_0071271, hsa_circ_0000284 and hsa_circ_0003344 (Table 1).

### The basic characteristics of the research object of Q-PCR experiment

To validate the candidate circRNAs, Q-PCR was conducted in the 103 pairs of subjects. There were 71 males and 32 females in the diabetes group with an age distribution of 50.71±12.57 years old and 71 males and 32 females in the control group with an age distribution of 50.22±12.68 years old. The distribution of age groups showed that the middle-aged group (45-59 years old) accounts for the largest proportion, followed by the youth group (18-44 years old), and the elderly group was less distributed in this study. Table 2 describes the demographic characteristics of the study subjects. Among them, occupational status and hyperlipidemia were statistically significant between the diabetes group and the control group, while the age group, smoking, drinking, and hypertension were not statistically different between the two groups.

Table 3 compares the expression levels of biochemical indexes between the diabetic group and the control group. Because the data distribution type was skewed, the Wilcoxon signed-rank test was used. Results showed that aspartate aminotransferase (AST), blood glucose (Glu), glycated hemoglobin (HbA1c), low-density cholesterol (LDL), triglyceride (TG), total cholesterol (TC), sodium (Na), chlorine (Cl), those indexes were statistically different between diabetes and the control group, while diastolic blood pressure (DBP), systolic blood pressure (SBP), alanine aminotransferase (ALT), high-density cholesterol (HDL), potassium (K), calcium (Ga), which was no statistically significant difference in the indexes between diabetes and the control group.

### Analysis of relative expression of target circRNA

The data processing of Q-PCR was analyzed by relative quantitative calculation method. The results showed that hsa_circ_0071106 had a statistically significant difference between the diabetic group and the control group, while hsa_circ_0000284, hsa_circ_0003344, and hsa_circ_0071271 had no statistically significant difference between the two groups. In addition, the ΔCt value of hsa_circ_0071106, hsa_circ_0071271, hsa_circ_0000284 from the control group to the diabetes group is decreasing, indicating that the expression level of hsa_circ_0071106, hsa_circ_0071271, hsa_circ_0000284 in the diabetes group was higher than that in the control group. The ΔCt value of hsa_circ_0003344 from the control group to the diabetes group was rising, that indicated the expression level of the diabetic group was lower than that of the control group, which was consistent with the results of the microarray analysis (Table 4).

### The diagnostic value of differentially expressed circRNA in diabetes

**Figure 4:**
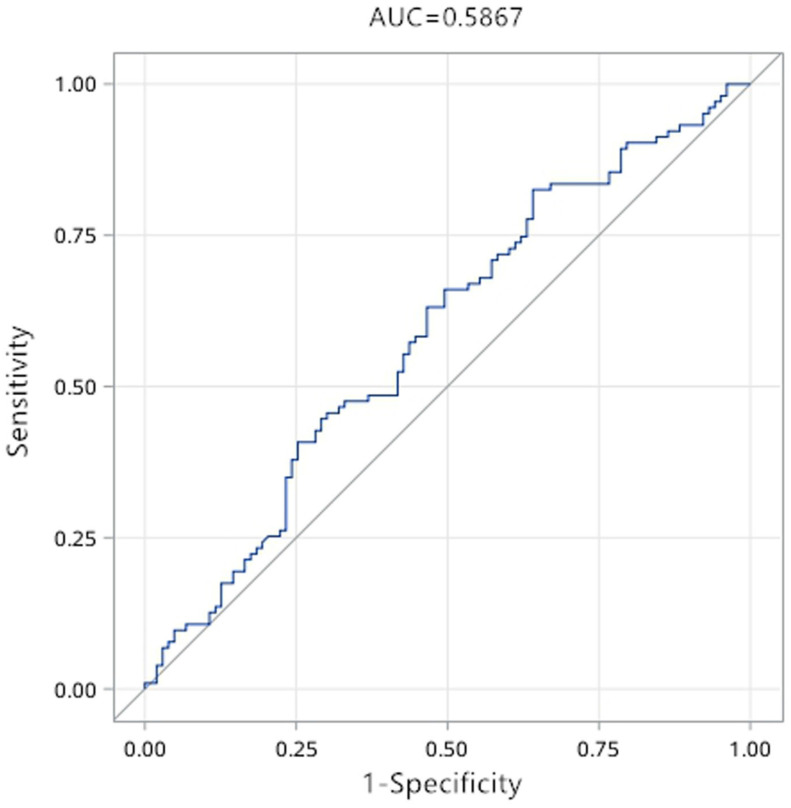

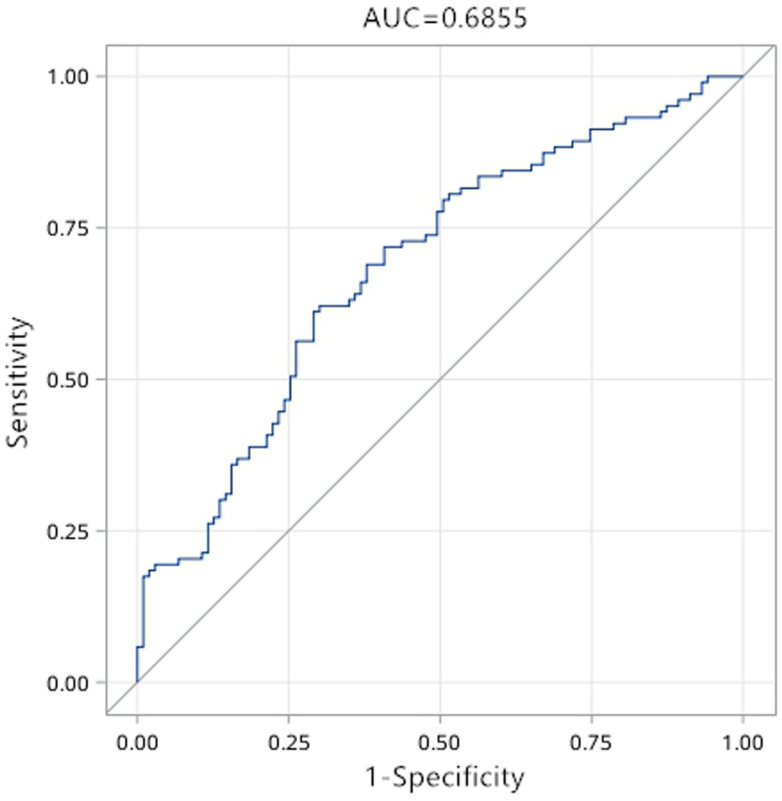
ROC curve of hsa_circ_0071106 in diabetes. Note: The picture on the top is the ROC curve before adjustment, and the picture on the bottom is the ROC curve after adjusting the influencing factors such as occupation, blood lipid (AUC<0.5 has no diagnostic value, AUC>0.5 is diagnostic value).

The results of Q-PCR verification showed that only the expression difference of hsa_circ_0071106 in diabetes among the selected candidate circRNAs had statistical significance. To determine the diagnostic values of hsa_circ_0071106 for type 2 diabetes, ROC curve analysis was performed (Figure 4). Using the grouping of diabetes as the state variable and the relative expression level of hsa_circ_0071106 as the test variable to draw the ROC curve. The results in Table 5 show that the sensitivity was 82.5%, the specificity was 35.9% and AUC was 0.587, suggesting that hsa_circ_0071106 have diagnostic value in the diagnosis of diabetes. According to the maximum Youden index, the best cutoff point for exploring has_circ_0071106 was 5.914. After adjusting occupation and blood lipid influencing factors, the AUC increased to 0.690, the sensitivity was 69.0%, and the specificity was 62.1%, and its diagnostic value was improved compared with that before adjusting the risk factors. According to the largest Youden index, the best cut-off point of hsa_circ_0071106 was 6.243. From the results, it could be seen that occupational, blood lipid and other factors should be adjusted for the differentially expressed circRNA, and a higher diagnosis accuracy and reliability could be obtained.

### Multivariate logistic regression analysis of hsa_circ_0071106 and diabetes

The factors that were meaningful in the analysis of the single-factor logistic regression model were incorporated into the multi-factor logistic regression model. After correction for occupation and blood lipids; table 6 showed the results of the multi-factor Logistic regression model, which indicated that occupation, hyperlipidemia and hsa_circ_0071106 had a stronger correlation with diabetes (*P*<0.05). The high expression level hsa_circ_0071106 was a risk factor and the high expression level of hsa_circ_0071106 was 2.819 (95%CI: 1.415~5.616) times higher than the low expression level of hsa_circ_0071106.

### Predict miRNA by circRNA

Import the raw data of hsa_circ_0071106 into miRNA target gene prediction software such as TargetScan and miRanda, predict five miRNAs that may be combined with hsa_circ_0071106. The results showed that the targets of miRNA bound to has_circ_0071106 were hsa-miR-6830-3p, hsa-miR-4743-3p, hsa-miR-2682-3p, hsa-miR-1206 and hsa-miR-29a-5p.

### hsa_circ_0071106 function prediction analysis

**Figure 5:**
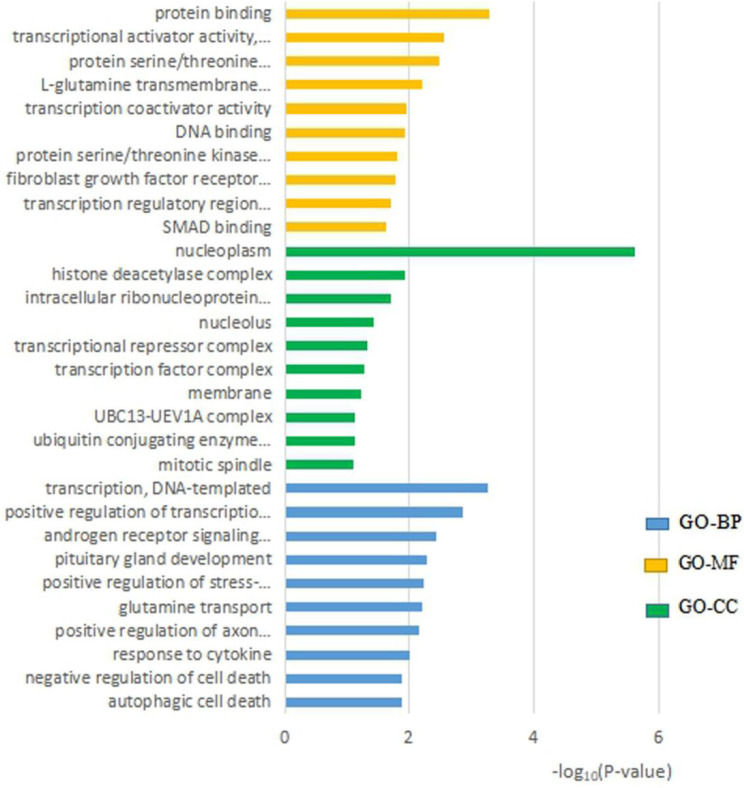
The top 10 significant GO entries of hsa_circ_0071106.

In order to predict the function of hsa_circ_0071106, we conducted a GO analysis. In the display of the results in Figure 5: the MF process showed that hsa_circ_0071106 was mainly enriched in the active elements of “protein binding”, the CC process was mainly enriched in cells such as “nucleus and cytoplasm”, and the BP process is mainly enriched in the process of “transcription, DNA template”.

### hsa_circ_0071106 path prediction analysis

In KEGG pathway analysis, the target genes of hsa_circ_0071106 were enriched with *P*<0.05. The results showed that the differentially expressed hsa_circ_0071106 was related to “MAPK signaling pathway” (Table 7).

## Discussion

This study revealed the expression levels of circRNAs in the peripheral blood of T2DM patients was significantly different from that of healthy subjects. A total of 798 circRNAs were detected, and the circRNA expression profile of diabetic blood samples was preliminarily constructed. According to the differential expression analysis, it was found that the expression levels of 86 circRNAs in diabetic group and healthy group were statistically significant. According to volcano and scatter plots, 70 circRNAs were up-regulated in diabetes, and 16 circRNAs were down-regulated. In these 86 circRNA differential expression profiles, we found that hsa_circ_0071106 had a statistically significant difference. To remove other influencing factors, the ROC curve showed that the AUC of hsa_circ_0071106 was 0.690. With a sensitivity of 62.1% and a specificity of 69.9%, showed that hsa_circ_0071106 could be considered as a biomarker for the diagnosis of T2DM.This result was consistent with Liu's.

However, what is the specific mechanism of action of hsa_circ_0071106 in T2DM? Through bioinformatics analysis technology, we use Targetscan, miRanda and other databases to predict five miRNA targets: hsa-miR-6830-3p, hsa-miR-4743-3p, hsa-miR-2682-3p, hsa-miR-1206 and hsa-miR-29a-5p, which were combined with the hsa_circ_0071106. Then the predicted target genes were analyzed by GO and KEGG pathway through DAVID online software, and the biological processes and possible signal pathways of hsa_circ_0071106 were preliminarily analyzed, which provided effective information for further research of hsa_circ_0071106.

Among the five miRNA targets predicted by hsa_circ_0071106, hsa-miR-29a-5p (miR-29a) has been verified by many cell models and animal experiments related to diabetes and complications. Wang Lei 21 studied that up-regulation of miR-29a in rat liver tissue caused down-regulation of FOXO3, which led to insulin resistance. Yang 22 revealed that miR-29a regulates the body's insulin secretion and glucose metabolism. MiR29a inhibits the expression of insulin receptor base 1 (IRS-1) during translation by acting on the 3'untranslated region (3'-UTR) of IRS-1. A large amount of reduced IRS-1 will damage the insulin signal transduction and glucose absorption of muscle cells. In addition, studies have confirmed that the decreased expression level of IRS-1 is involved in the pathogenesis of T2DM 23-25. Aibin He 26 found that the presence of miRNA-29a/miRNA-29b in adipocytes would impede the absorption of glucose. When miRNA-29 is at a high level, it will inhibit the activation of Akt and cause insulin resistance. At this time, the internal environment is in High sugar levels, and the high sugar internal environment will promote the expression of miRNA-29a/miRNA-29b, making the condition of diabetes worse. Therefore, studies have shown that hsa-miR-29a-5p (miR-29a) may affect the pathogenesis of diabetes through the way of causing insulin resistance. The enrichment results of GO analysis showed that hsa_circ_0071106 may mainly exist in the nucleoplasm, bind to a certain protein, or participate in the process of transcribed genes. The KEGG pathway results show that hsa_circ_0071106 with differential expression is related to the MAPK signaling pathway, which is an important pathway in the cell signal transduction system 27 and one of the pathways that affect insulin signaling 28. The MAPK signaling pathway includes four extracellular signal pathways including extracellular signal-regulated kinase (ERK), p38 mitogen-activated protein kinase (p38MAPK), jnk amino terminal kinase (JNK), and ERK5/BMK1 27. ERK, p38MAPK, and JNK signaling pathways are involved in regulating insulin transduction. The ERK signaling pathway is involved in the regulation ofIRS-1. Liu 29 and others have studied that insulin sensitivity is affected by the activity of ERK1/2MAPKs. When the activity increases, insulin sensitivity decreases. Yoo 30 found that the JNK signaling pathway promoted phosphorylation of the PTB segment S307 of IRS-1. The role of the p38MAPK signaling pathway in diabetes was different from that of ERK and JNK. The p38MAPK signaling pathway could increase the expression of glucose transporter 4 (GluT4) proteins, and the smooth transport of GluT4 could reduce the body's insulin resistance or T2DM. However, recent studies have also found that p38MAPK could accelerate cell proliferation and increase protein deposition under the action of high glucose and transforming factor (TGF-β), which could lead to complications such as diabetic nephropathy or diabetic vascular disease. This study found that the expression level of hsa_circ_0071106 was higher in T2DM group, so the overexpression of hsa_circ_0071106 may play a role in promoting the onset of diabetes. In addition, after the enrichment of the KEGG pathway, it has been shown that hsa_circ_0071106 is related to the transduction of the MAPK signaling pathway. It can be speculated that hsa_circ_0071106 may be involved in the process of JNK-mediated phosphorylation of IRS-1, or may be involved in the process of ERK regulating IRS-1; which in turn affects blood glucose metabolism balance. To sum up, the function prediction results showed that hsa_circ_0071106 was involved in biological processes such as protein binding or regulation of gene transcription, or MAPK signaling pathway that regulates insulin signal transduction, or may be involved in the pathway of hsa-miR-29a-5p regulating diabetes.

The limitations of this study were as follows: 1. in this study, only 4 circRNAs were selected from the circRNA differential expression profiles for Q-PCR verification of the population. For other differentially expressed circRNAs, no further population verification was done. 2. The bioinformatics analysis in this study only predicts the functional mechanism of circRNA with differential expression, and has not been verified by cell and animal experiments. 3. This study was a single-center study. The living environment of the research subjects was the same. Whether the circRNA gene expression profile was applicable to other populations was unclear. Therefore, further research could be conducted by collecting people from different regions for verification.

## Figures and Tables

**Table 1:** Primer sequences of target circRNA and GAPDH

Primers	Amplified sequence
hsa_circ_0071271-F	5'-GGAACCCAAAGACCTGCTACAA-3'
hsa_circ_0071271-R	5'-TGGTCCACTCCAGCTCCTCT-3'
hsa_circ_0003344-F	5'-GGAAGAGCTACGGGAGATCAA-3'
hsa_circ_0003344-R	5'-CCAGGTCTCCCTTATCGACCT-3'
hsa_circ_0000284-F	5'-ATAGACTTTGGGTCGGCCAGT-3'
hsa_circ_0000284-R	5'-TCTTCACACTACAAAAGGCACTTGA-3'
hsa_circ_0071106-F	5'-GAAGCTGCTGATCGGAAGAAA-3'
hsa_circ_0071106-R	5'-GCCGGTTCTGCTCTACTTGG-3'
GAPDH-F	5'-GCACCGTCAAGGCTGAGAAC-3'
GAPDH-R	5'-TGGTGAAGACGCCAGTGGA-3'

**Table 2:** Demographic characteristics of the research object

Variable	Control (n=103)	T2DM (n=103)	χ^2^	*P*
**Age**			0.047	0.977
18~	31 (30.10)	30 (29.13)		
45~	56 (54.37)	56 (54.37)		
60~	16 (15.53)	17 (16.50)		
**Occupation**			14.842	0.002
Unemployment	34 (33.01)	60 (58.25)		
Business people	7 (6.8)	3 (2.91)		
Farmer	11 (10.68)	11 (10.68)		
Industrialist	51 (49.51)	29 (28.16)		
**Smoke**			0.951	0.330
No	75 (72.82)	81 (78.64)		
Yes	28 (27.18)	22 (21.36)		
**Drink**			0.126	0.723
No	86 (83.5)	87 (85.29)		
Yes	17 (16.5)	15 (14.71)		
**Hyperlipidemia**			9.690	0.002
No	91 (55.49)	12 (28.57)		
Yes	73 (44.51)	30 (71.43)		
**Hypertension**			1.215	0.270
No	79 (52.32)	24 (43.64)		
Yes	72 (47.68)	31 (56.36)		

**Table 3:** Comparison of the expression levels of biochemical indexes between the diabetes group and the control group

Variable	Control	T2DM	*Z*	*P*
SBP (mmHg)	134 (123,151)	138 (125,157)	274.0	0.370
DBP (mmHg)	82 (74,94)	85 (75,94)	201.0	0.486
ALT (IU/L)	20 (15,38)	24 (17,39)	23.5	0.936
AST (IU/L)	20.6 (17,31)	19 (15.2,23)	-931.5	0.002
Glu	5.6 (5.2,6.2)	15.8 (10.8,22.5)	2516.5	<0.001
HbA1c	5.4 (5.1,5.7)	10.4 (9.0,11.9)	2678.0	<0.001
LDL (mmol/L)	2.89 (2.53,3.29)	3.22 (2.74,3.86)	862.5	0.004
HDL (mmol/L)	1.36 (1.19,1.50)	1.37 (1.21,1.56)	388.0	0.197
TG (mmol/L)	1.45 (0.98,3.64)	2.82 (2.10,4.65)	1795.0	<0.001
TC (mmol/L)	4.60 (4.04,5.20)	5.39 (4.80,6.10)	1278.0	<0.001
K	3.89 (3.68,4.14)	3.97 (3.69,4.16)	233.5	0.438
Ca	2.33 (2.24,2.41)	2.35 (2.24,2.40)	-10.0	0.972
Na	138.90(135.20,141.00)	134.10(128.20,137.60)	-1050.5	<0.001
Cl	103.50 (101.5,106.0)	99.5 (98.0,101.9)	-2060.0	<0.001

**Table 4:** Verification of relative expression of target circRNA in diabetes and control group by qPCR

Target circRNA	Control	T2DM	*t*	*P*
hsa_circ_0071106	5.58±0.75	5.34±0.71	2.65	0.009
hsa_circ_0000284	2.35±0.75	2.29±0.65	0.66	0.508
hsa_circ_0003344	4.25±1.29	4.30±1.17	0.32	0.751
hsa_circ_0071271	8.50±1.39	8.43±1.32	0.56	0.577

**Table 5:** Diagnostic value of differentially expressed circRNA in diabetes

circRNA	AUC	sensitivity	specificity	Cut off	Youden
hsa_circ_0071106 (After)	0.587	0.825	0.359	5.914	0.184
hsa_circ_0071106 (Later)	0.690	0.621	0.699	6.243	0.320

**Table 6:** Multivariate logistic regression of hsa_circ_0071106 and diabetes

Variable	*β*	S	*z*	*P*	OR (95% CI)	After OR (95% CI)
**Occupation**						
No	1.000	-	-	-	-	-
Yes	-1.030	0.307	11.268	<0.001	0.350 (0.190~0.643)	0.357 (0.196~0.651)
**Hyperlipidemia**						
No	1.000	-	-	-	-	-
Yes	0.948	0.396	5.741	0.017	2.800 (1.360~5.764)	2.581 (1.188~5.604)
**hsa_circ_0071106**						
Low	1.000	-	-	-	-	-
High	1.036	0.352	8.687	0.003	2.478 (1.306~4.701)	2.819 (1.415~5.616)

**Table 7:** KEGG pathway enrichment analysis of hsa_circ_0071106

KEGG signaling pathway	Number of genes (%)	*P*
MAPK signaling pathway	12 (2.4)	0.039
Melanoma	7 (1.8)	6.7×10^-3^
Osteoclast differentiation	7 (1.4)	9.2×10^-3^
Mucin type O-Glycan biosynthesis	4 (0.8)	0.037
